# Early stimulated thyroglobulin for response prediction after recombinant human thyrotropin-aided radioiodine therapy

**DOI:** 10.1007/s12149-017-1190-3

**Published:** 2017-07-07

**Authors:** Hee Jeong Park, Jung-Joon Min, Hee-Seung Bom, Jahae Kim, Ho-Chun Song, Seong Young Kwon

**Affiliations:** 1 Department of Nuclear Medicine, Chonnam National University Hwasun Hospital, 322 Seoyang-ro, Hwasun-eup, Hwasun-Gun, Jeonnam 58128 South Korea; 2 Department of Nuclear Medicine, Chonnam National University Hospital, 42 Jebong-ro, Donggu, Gwangju, 61469 South Korea

**Keywords:** Differentiated thyroid carcinoma, Radioiodine therapy, Recombinant human thyrotropin, Thyroglobulin, Therapeutic response

## Abstract

**Objective:**

Measurement of recombinant human thyrotropin (rhTSH)-stimulated thyroglobulin (Tg) is generally recommended 72 h after the second rhTSH injection. However, due to the acute effect of I-131 on thyrocytes, Tg measured after radioiodine therapy (RIT) would not accurately reflect the thyroid tissue burden. We aimed to determine predictive values of serum Tg level measured just before rhTSH-aided RIT and to compare the results obtained just after RIT in patients with differentiated thyroid carcinoma (DTC).

**Methods:**

We evaluated 150 patients with DTC who underwent rhTSH-aided RIT (2.96–6.66 GBq) after total thyroidectomy between 2009 and 2014. Serum Tg level was measured 24 h (early Tg) and 72 (or 96) h (delayed Tg) after the second rhTSH injection. An excellent response was defined based on the latest American Thyroid Association Guidelines. Univariate and multivariate analyses were performed for early Tg, delayed Tg, and other clinical variables.

**Results:**

In the multivariate analysis, tumor size [odds ratio (OR) 1.716; 95% confidence interval (CI) 1.019–2.882; *p* = 0.042] and early Tg level (OR 2.012; 95% CI 1.384–2.925, *p* < 0.001) independently predicted excellent responses. The cutoff for the best early Tg level to predict a non-excellent response was 2.0 ng/mL. Delayed Tg was not a significant predictor (OR 0.992; 95% CI 0.969–1.015; *p* = 0.492).

**Conclusions:**

Early stimulated Tg significantly predicted therapeutic response after rhTSH-aided RIT in patients with DTC. Therefore, serum Tg should be measured before RIT to predict therapeutic responses.

## Introduction

Total thyroidectomy followed by radioiodine therapy (RIT) is the treatment of choice for differentiated thyroid carcinoma (DTC) [1, 2]. Although DTC shows a relatively good response to RIT, unsuccessful ablation is reported in about 6–30% of patients [3–8], so additional treatments, such as surgical procedures or repeated RIT, and life-long follow-up are required. Serum thyroglobulin (Tg) levels measured during hypothyroidism, just before RIT, have good prognostic values for predicting successful ablation [9–12].

Recombinant human thyrotropin (rhTSH) represents a safe and effective means of increasing serum TSH in preparation for RIT, avoiding signs and symptoms of hypothyroidism associated with thyroid hormone withdrawal (THW) [13, 14]. I-131 is usually administered on the day after the 2nd injection of rhTSH according to the conventional schedule [5, 6]. On the other hand, serum Tg should be measured 72 h after final injection of rhTSH, because this day corresponds to the peak serum Tg levels [15, 16]. However, due to the radioiodine-induced damage to normal thyroid cells and release of stored Tg in thyroid follicles, the Tg level measured after RIT would not be reliably predictive [17, 18].

The present study was undertaken to determine the value of Tg measured before and after RIT that predicts a therapeutic response in DTC patients with rhTSH-aided RIT.

## Materials and methods

### Subjects

Between August 2009 and May 2014, 430 DTC patients, who underwent total or near-total thyroidectomy followed by rhTSH-aided RIT in our institution, were initially analyzed. Among these patients, patients with high levels of serum anti-Tg autoantibodies (TgAbs) more than 100 IU/mL (*n* = 37), repeated RIT (*n* = 27), missing Tg measurements (*n* = 109), distant metastases (*n* = 8), and insufficient follow-up (*n* = 99) were excluded. Finally, 150 patients were enrolled in this study. This retrospective study was performed in accordance with the ethical standards laid down in the 1964 Declaration of Helsinki and its later amendments, and ethical approval was obtained from the local ethics committee (CNUHH-2016-069). For this type of study, informed consent was waived.

### Radioiodine therapy

Patients were referred for RIT 0–27 months (mean 4.0 months) after surgery. All patients had received 2 consecutive daily doses of 0.9 mg rhTSH (Thyrogen, Genzyme Transgenics Corp., Cambridge, USA) administered by the intramuscular route prior to RIT. According to the instructions regarding limiting exposure to environmental iodine, patients consumed a low-iodine diet for 2 weeks prior to RIT. Twenty-four hours after the 2nd injection of rhTSH, a therapeutic dose of I-131 (2.96–6.66 GBq) was administered. An I-131 whole body scan was performed 7–8 days after oral ingestion of I-131.

### Tg and TSH measurement

For serum Tg measurements, blood samples were collected two times: before RIT (24 h after the 2nd rhTSH injection) (early Tg) and after RIT (72 or 96 h after the 2nd rhTSH injection) (delayed Tg). Serum Tg was measured by an immunoradiometric assay (IRMA) (RIA Tg-plus, BRAHMS GmbH, Hennigsdorf, Germany) with an analytical sensitivity (AS) and functional sensitivity (FS) of 0.08 and 0.2 ng/mL, respectively. Serum TgAbs was measured by a radioimmunoassay method (RIA anti-Tgn, BRAHMS GmbH, Hennigsdorf, Germany) with an AS of 5.5 U/mL and FS of <20 U/mL. Serum TSH levels were determined by IRMA (TSH-CTK-3, DiaSorin, Saluggia, Italy) with an AS and FS of 0.04 and 0.07 mU/L, respectively. The cut-off point for TSH, above which corresponding stimulated Tg levels were acceptable, was 30 mU/L.

### Follow-up examination

The first post-ablation follow-up examination was performed 6–12 months (mean 9.0 months) after RIT. The follow-up protocol included neck ultrasound (US) and serum Tg measurement. Suppressed Tg was measured in 60 patients, and the remainder of the patients (*n* = 90) underwent diagnostic I-123 whole body scan (DxWBS) with stimulated Tg aided by rhTSH or endogenous TSH.

Definitions of therapeutic responses followed the 2015 American Thyroid Association Guidelines [19]: (1) excellent response—negative imaging and either suppressed Tg <0.2 ng/mL or TSH-stimulated Tg <1 ng/mL; (2) biochemical incomplete response—negative imaging and suppressed Tg ≥1 ng/mL or stimulated Tg ≥10 ng/mL; (3) structural incomplete response—structural or functional evidence of disease with any Tg level with or without TgAbs; (4) indeterminate response—nonspecific findings on imaging studies or suppressed Tg detectable but <1 ng/mL or stimulated Tg detectable but <10 ng/mL. We classified patients into 2 groups: the excellent response group and the non-excellent response group (which included those showing a biochemical incomplete response, structural incomplete response, and indeterminate response).

### Statistical analysis

Using a logistic regression analysis, clinicopathologic variables were tested for their association with an excellent response using a univariate analysis: early Tg, delayed Tg, other clinical (age at diagnosis, sex, I-131 dose), and pathologic markers [American Joint Cancer Committee (AJCC)/International Union Against Cancer (UICC) TNM staging (7th edition), presence of extra-thyroid extension, tumor size, multicentricity, and bilaterality]. Variables with *p* value ≤0.20 were further analyzed by a multivariate logistic regression analysis using the stepwise backward method [20, 21]. A *p* value <0.05 was considered statistically significant. A receiver-operating characteristic (ROC) curve analysis was used to define the best cut-off value for serum Tg at ablation. For the established cut-off value, we calculated the sensitivity, specificity, positive predictive value (PPV), negative predictive value (NPV), and area under the curve (AUC). Statistical analysis was performed using SPSS version 21.0 for Windows^®^ (IBM Corp., Armonk, USA).

## Results

Patients’ characteristics are summarized in Table 1. The study population comprised 114 women (76.0%) and 36 men (24.0%), aged 20–79 years (mean 48.8) at the time of surgery. The histological DTC subtype was papillary in 148 cases (98.7%) and follicular in 2 cases (1.3%). At the time of RIT, early Tg ranged from 0.1 to 117.9 ng/mL (mean 3.2) and delayed Tg ranged from 0.1 to 198.7 ng/mL (mean 9.6).

**Table 1:** Patients’ characteristics

Parameter	No. of patients (%)
Age (years)	48.8 ± 11.8 (range 20–79)
Sex	
Male	36 (24.0%)
Female	114 (76.0%)
T stage	
T1	85 (56.7%)
T2	6 (4.0%)
T3	48 (32.0%)
T4	11 (7.3%)
N stage	
N0/Nx	29 (19.3%)
N1a	95 (63.3%)
N1b	26 (17.3%)
Tumor size (cm)	1.1 ± 0.7 (range 0.3–5.0)
Multiplicity	
No	69 (46.0%)
Yes	81 (54.0%)
Bilaterality	
No	90 (60.0%)
Yes	60 (40.0%)
Extrathyroidal extension	
No	91 (60.7%)
Yes	59 (39.3%)
Tumor pathology	
Papillary	148 (98.7%)
Follicular	2 (1.3%)
Dose of administered I-131 (GBq)	
2.96	18 (12.0%)
3.70	87 (58.0%)
5.55	9 (6.0%)
6.66	36 (24.0%)
Early Tg (ng/mL)	3.2 ± 11.4 (range 0.1–117.9)
Delayed Tg (ng/mL)	9.6 ± 27.1 (range 0.1–198.7)
Early TSH (mU/L)	94.9 ± 23.7 (range 46.4–166.9)
Delayed TSH (mU/L)	31.4 ± 27.5 (range 2.8–80.5)

*Early Tg and early TSH* serum Tg and TSH measured just before radioiodine therapy, *delayed Tg and delayed TSH* serum Tg and TSH measured after radioiodine therapy, *Tg* thyroglobulin, *TSH* thyrotropin

At the follow-up examination, an excellent response was observed in 85 (56.7%) patients. In the 65 (43.3%) patients with non-excellent responses, 10 (6.7%) patients had a biochemical incomplete response, 10 (6.7%) had a structural incomplete response, and 45 (30.0%) had an indeterminate responses.

Variables significantly associated with an excellent response in the univariate analysis are reported in Table 2. Older age (*p* = 0.041), smaller tumors (*p* = 0.009), and low level of early Tg (*p* < 0.001) significantly predicted an excellent response. In the multivariate logistic regression analysis, early Tg [odds ratio (OR) 2.012; 95% confidence interval (CI) 1.384–2.925; *p* < 0.001] and tumor size (OR 1.714; 95% CI 1.019–2.882; *p* = 0.042) significantly predicted an excellent response (Table 3). However, delayed Tg did not show statistical significance for response prediction after RIT (OR 0.992; 95% CI 0.969–1.015; *p* = 0.492).

**Table 2:** Results of the univariate analysis of therapeutic response prediction-related parameters

Parameters	Excellent response, *n* (%)	Non-excellent response, *n* (%)	*p* value
Age (years)			
<45 years	24 (45.3%)	29 (54.7%)	0.041*
≥45 years	61 (62.9%)	36 (37.1%)	
Sex			
Male	18 (50.0%)	18 (50.0%)	0.441
Female	67 (58.8%)	47 (41.2%)	
T stage			
T1	52 (61.2%)	33 (38.8%)	0.274
T2	2 (33.3%)	4 (66.7%)	
T3	27 (56.3%)	21 (43.8%)	
T4	4 (36.4%)	7 (63.6%)	
N stage			
N0/Nx	19 (65.5%)	10 (34.5%)	0.205
N1a	55 (57.9%)	40 (42.1%)	
N1b	11 (42.3%)	15 (57.7%)	
Tumor size (cm)	1.0 ± 0.6	1.3 ± 0.9	0.009*
Multiplicity			
No	37 (53.6%)	32 (46.4%)	0.512
Yes	48 (59.3%)	33 (40.7%)	
Bilaterality			
No	48 (53.3%)	42 (46.7%)	0.401
Yes	37 (61.7%)	23 (38.3%)	
Extrathyroidal extension			
No	54 (59.3%)	37 (40.7%)	0.500
Yes	31 (52.5%)	28 (47.5%)	
Dose of administered I-131 (GBq)			
2.96	13 (72.2%)	5 (27.8%)	0.412
3.70	48 (55.2%)	39 (44.8%)	
5.55	6 (66.7%)	3 (33.3%)	
6.66	18 (50.0%)	18 (50.0%)	
Early Tg (ng/mL)	0.8 ± 0.8	6.4 ± 16.8	<0.001*
Delayed Tg (ng/mL)	5.3 ± 16.2	14.9 ± 36.0	0.074^†^

*Tg* thyroglobulin

* Statistically significant (*p* < 0.05)

^†^Statistical trend (0.05 ≤ *p* ≤ 0.20)

**Table 3:** Results of the multivariate analysis of therapeutic response prediction-related parameters

Parameter	Odds ratio (95% CI)	*p* value
Age (years)	0.450 (0.202–0.999)	0.050
Tumor size (cm)	1.714 (1.019–2.882)	0.042*
Early Tg (ng/mL)	2.012 (1.384–2.925)	<0.001*
Delayed Tg (ng/mL)	0.992 (0.969–1.015)	0.492

*CI* confidence interval, *Tg* thyroglobulin

* Statistically significant (*p* < 0.05)

**Fig. 1:**
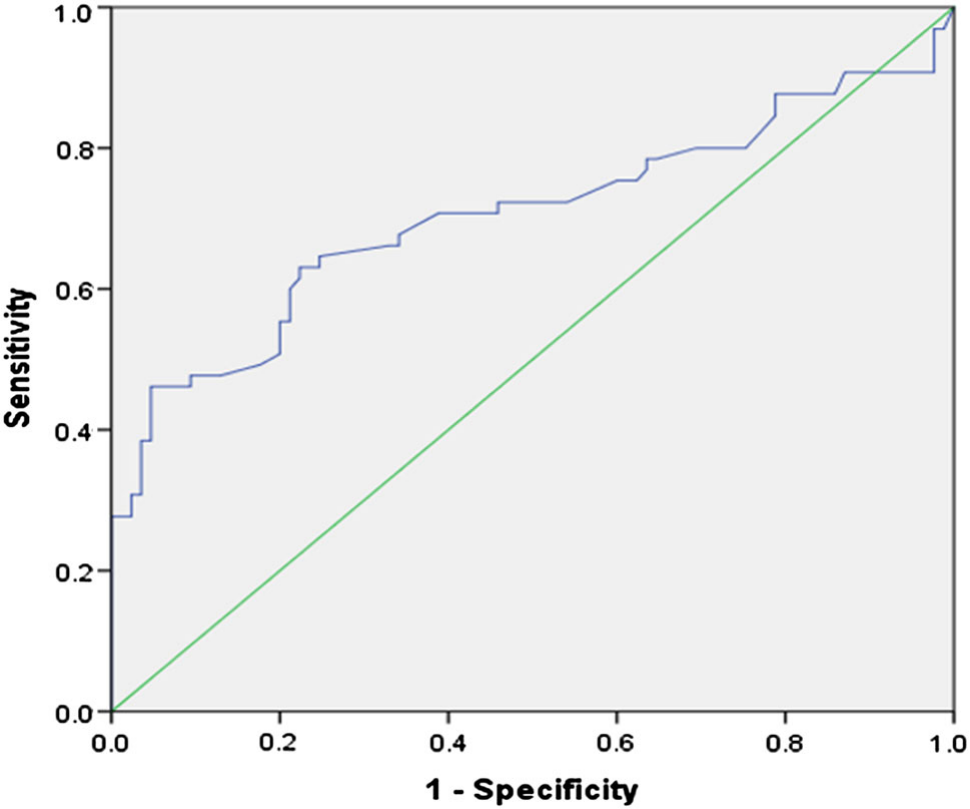
Results of the receiver-operating characteristic (ROC) curve analysis for the early thyroglobulin levels that predict non-excellent responses after rhTSH-aided radioiodine therapy

An ROC curve analysis showed that the optimal cut-off value for early Tg was 2.0 ng/mL; it had a sensitivity of 46.2%, specificity of 95.3%, PPV of 88.2%, and NPV of 69.8% for predicting a non-excellent response (AUC 0.704; *p* < 0.001) (Fig. 1). In 116 patients with an early Tg level ≤2.0 ng/mL, an excellent response was observed in 81 patients (69.8%), whereas only 4/34 (11.8%) patients with early Tg levels >2.0 ng/mL showed an excellent response (Table 4).

**Table 4:** Early Tg levels and percentages of patient responses to RIT

Early Tg levels (ng/mL)	Excellent response, *n* (%)	Biochemical incomplete response, *n* (%)	Structural incomplete response, *n* (%)	Indeterminate response, *n* (%)	Total, *n* (%)
≤2.0	81 (69.8%)	1 (0.9%)	2 (1.7%)	32 (27.6%)	116 (100%)
>2.0	4 (11.8%)	9 (26.5%)	8 (23.5%)	13 (38.2%)	34 (100%)

*Tg* thyroglobulin, *RIT* radioiodine therapy

## Discussion

In patients with DTC, the stimulated Tg level measured just before RIT proved to be a biochemical tumor marker that could predict persistent or recurrent disease after THW [9–12]. In a recent meta-analysis involving 3947 patients across a broad spectrum of disease, it was demonstrated that the pre-ablation Tg level could be a useful negative predictor of persistent or recurrent DTC. The overall NPV was 94% when the pre-ablation Tg level was <10 ng/mL [12]. In addition, the stimulated Tg measured after THW could be used to predict the volume of the thyroid remnants or the residual tumor burden [22]. However, there is no consensus available with respect to the clinical implications or testing protocol for stimulated Tg at rhTSH-aided RIT, although rhTSH is now widely used as a method for RIT preparation.

From the previous studies, serum Tg should be measured in the 3 days after the 2nd injection of rhTSH [15, 16]. In 1998, Genzyme showed the serial Tg data that resulted in FDA approval. In that study protocol, 24 h after the 2nd rhTSH injection, 148 MBq radioiodine were administered orally and Tg levels were measured on days 1, 2, 3, and 7. They found that maximum serum Tg levels were observed on day 3 in most patients. However, the administered dose of radioiodine was not a therapeutic dose that could cause acute thyroid injury [16]. This timing of Tg measurement has raised concerns about its reliability as a tumor marker, because acute thyroid injuries would contribute to Tg production. Taieb et al. [17] showed sequential elevation of median serum Tg levels during the 48 h after I-131 administration (3.7 GBq) in DTC patients with hypothyroidism. Tg levels increased due to an acute effect of I-131 on Tg release. Furthermore, the Tg increments at 48 h were not correlated with Tg levels measured before I-131 administration.

There have been inconsistent studies to show the prognostic value of early Tg and delayed Tg in DTC patients prepared with rhTSH. Ciappuccini et al. [23] showed a significant correlation between the rhTSH-stimulated Tg levels measured immediately before RIT and persistent/recurrent disease (PRD), defined as evidence of tumor burden confirmed by histology or radiological modalities on follow-up. The early Tg levels were significantly lower in nonstructural (microscopic) than in structural (macroscopic) residual disease. The reported Tg cut-off value for predicting PRD was 2.8 ng/mL, closed to the threshold defined in our study, with a sensitivity of 86%, specificity of 64%, PPV of 24%, and NPV of 97%. On the other hand, Melo et al. [24] demonstrated that rhTSH-stimulated Tg measured 3 days after RIT had a predictive value for disease persistence or recurrence 1 year later in 131 consecutive patients. From this study, Tg levels ranged from 0 to 1927 ng/mL and were significantly lower in the disease-free group (17.9 ± 49.1 vs 136.3 ± 341.0 ng/mL, *p* < 0.001), with an optimal Tg cut-off level of 7.2 ng/mL. However, there was no previous report to compare the prognostic value of early Tg and delayed Tg.

Our study showed that rhTSH-stimulated Tg level just before RIT (early Tg) was an independent predictor of excellent response on follow-up, whereas there was no significant difference in Tg levels after RIT (delayed Tg) between the excellent and non-excellent response groups (5.3 ± 16.2 vs 14.9 ± 36.0, *p* = 0.074). Several explanations could be possible. Compared to the study by Melo et al., the proportion of patients with structural incomplete responses among the non-excellent responders were very low in our study (80.0 vs 15.4%). The Tg levels could also reflect the proportion of structural incomplete responses in a group of patients with non-excellent responses between the two studies. Therefore, the Tg level after RIT (delayed Tg) might not have a predictive value for a group of patients with a low proportion of structural incomplete responses, where acute injury effects of I-131 on Tg release could affect the total level of Tg to evaluate remnant tissue burden.

Giovanella et al. [25] suggested that even Tg measured before TSH stimulation can reflect expression of thyroid remnants by showing a significant relationship between neck radioiodine uptake measured just before ablation and Tg level measured before rhTSH stimulation and under T4 treatment. Our study showed that Tg cutoff at 2.0 ng/mL provided high specificity (95.3%) and PPV (88.2%) for predicting a non-excellent response, which could be very helpful to decide follow-up strategy based on early Tg. However, the early Tg still has several limitations as predictive factor due to the short TSH stimulation time. In our study, Tg cutoff at 2.0 ng/mL provided insufficient sensitivity (46.2%) and NPV (69.8%). Tg cut-off value under short TSH stimulation could affect diagnostic accuracy. Our results also indicated that 30.2% of patients with Tg levels ≤2.0 ng/mL could not achieve an excellent response. Most of them belong to an indeterminate response group (Table 4), which of 13–20% are reclassified as having PRD over approximately 10 years of follow-up [26]. Further investigations are necessary to overcome the physiologic drawbacks of early Tg as a reliable predictive marker.

There are several limitations in our study. First, this study was retrospective, so a selection bias was inevitable. Especially, our protocol for I-131 dose selection has changed over the years based on recent studies and guidelines, which the choice of administered dose was heterogeneous, although there was no significant difference in therapeutic response among I-131 dose like previous studies (Table 2) [5, 6, 19]. Second, delayed Tg was measured 96 h after the 2nd injection of rhTSH in some patients due to their admission schedules. However, between the two patient groups undergoing Tg testing in 72 or 96 h, there was no significant difference in the delayed Tg (9.8 ± 29.7 vs 8.8 ± 21.0 ng/mL, *p* = 0.832) and delayed TSH (34.1 ± 27.1 vs 28.0 ± 28.1 mU/L, *p* = 0.202) levels. Third, although assessment of therapeutic responses was based on the latest ATA guidelines, follow-up protocols were not consistent for all patients. Some patients underwent DxWBS with stimulated Tg, and some patients underwent neck US with suppressed Tg. Further large-scale prospective studies are required to better determine the significance of early Tg.

In conclusion, the early stimulated serum Tg just before rhTSH-aided RIT significantly predicted therapeutic responses after RIT in patients with DTC. Therefore, physicians should consider measuring the serum Tg level before RIT to predict the therapeutic response, although the early Tg does not precisely reflect the remnant thyroid tissue burden in DTC patients prepared with rhTSH.
